# “Five hundred years of medicine gone to waste”? Negotiating the implementation of an intercultural health policy in the Ecuadorian Andes

**DOI:** 10.1186/s12889-018-5601-8

**Published:** 2018-06-04

**Authors:** Ana Llamas, Susannah Mayhew

**Affiliations:** London School of Hygiene and Tropical Medicine, Faculty of Public Health and Policy, 15-17 Tavistock place, London, WC1H 9SH UK

**Keywords:** Health policy, Maternal health, Indigenous health, Intercultural health, Policy implementation

## Abstract

**Background:**

In Ecuador, indigenous women have poorer maternal health outcomes and access to maternity services. This is partly due to cultural barriers. A hospital in Ecuador implemented the Vertical Birth (VB) policy to address such inequities by adapting services to the local culture. This included conducting upright deliveries, introducing Traditional Birth Attendants (TBAs) and making physical adaptations to hospital facilities.

**Methods:**

Using qualitative methods, we studied the VB policy implementation in an Ecuadorian hospital to analyse the factors that affect effective implementation of intercultural health policies at the local level. We collected data through observation, in-depth interviews, a focus group discussion, and documentation review. We conducted 46 interviews with healthcare workers, managers, TBAs, key informants and policy-makers involved in maternal health. Data analysis was guided by grounded theory and drew heavily on concepts of “street-level bureaucracy” to interpret policy implementation.

**Results:**

The VB policy was highly controversial; actors’ values (including concerns over patient safety) motivated their support or opposition to the Vertical Birth policy. For those who supported the policy, managers, policy-makers, indigenous actors and a minority of healthcare workers supported the policy, it was critical to address ethnic discrimination to improve indigenous women’s access to the health service. Most healthcare workers initially resisted the policy because they believed vertical births led to poorer clinical outcomes and because they resented working alongside TBAs. Healthcare workers developed coping strategies and effectively modified the policy. Managers accepted these as a compromise to enable implementation.

**Conclusions:**

Although contentious, intercultural health policies such as the VB policy have the potential to improve maternity services and access for indigenous women. Evidence-base medicine should be used as a lever to facilitate the dialogue between healthcare workers and TBAs and to promote best practice and patient safety. Actors’ values influenced policy implementation; policy implementation resulted from an ongoing negotiation between healthcare workers and managers.

## Background

Ecuador is making progress towards improving maternal health [49] although significant disparities remain between different ethnic groups [15, 36]. Data are not routinely disaggregated by ethnic origin but some reports show that Maternal Mortality Rate (MMR) is higher among indigenous women (117 per 1000,000 live births) than white women (45 per 100,000 live births) [36, 38]. Process indicators confirm ethnic inequities in reproductive health; for example, the use of institutional delivery and skilled birth attendants, two key indicators in maternal survival [22], are significantly lower amongst indigenous women compared with non-indigenous women [8, 9].

Traditionally, indigenous women in the study setting have given birth at home, looked after by Traditional Birth Attendants (TBAs) who provided one-to-one respectful care throughout pregnancy and postnatal period [26]. Other researchers have reported similar poor experiences of maternity services among indigenous women, who feel discriminated against and find maternity services unacceptable due to the lack of quality of care, and respect for their culture and birthing practices [3, 7, 34].

As a result of disparities in health outcomes and indigenous women’s poor experiences in health services, there are persistent calls to make health services responsive to the particular needs of indigenous women [8, 9, 19, 28, 30, 33, 38]. Across Latin America countries are developing intercultural health policies which encompass both indigenous traditional and western medicine and aim to increase access to services and reduce ethnic health inequities [31, 32].

In our study setting, the local hospital implemented one such policy, the Vertical Birth (VB) policy, which involved building a room adjacent to the labour ward resembling an indigenous house and a maternity waiting house. The room was equipped with ropes and bars that allowed women to adopt vertical or upright positions during delivery, which was considered a critical factor in traditional indigenous birth practices. Efforts were made to maintain women’s modesty by providing them with appropriate gowns and installing curtains in the dilatation room. Women were allowed to eat and drink during labour, choose their birthing position and have a birth companion. Early breastfeeding and bonding with the neonate were promoted. In addition, healthcare workers (HWs) received lessons in Kichwa (the local indigenous language), indigenous culture and training on clinical issues such as delivering women in upright positions. TBAs were also incorporated into the labour ward. The TBAs’ role was to support women and provide traditional care (e.g. massage, herbal remedies) throughout labour in collaboration with health professionals [34].

In spite of the growing popularity of intercultural health policies and the roll-out of the VB policy across Ecuador, there is little research thus far on the processes or impact of these policies. The few studies that have been conducted show mixed results. In Peru following the introduction of a similar policy to the VB policy and changes to strengthen health services, research found a sharp increase in facility deliveries [10] and met need for emergency obstetric care [21]. Research in other Latin American countries has found that potential health gains are compromised by implementation constraints due to tensions between HWs and TBAs, and lack of community involvement [43, 44, 46, 47].

## Methods

### Theoretical framework

The policy implementation literature shows that tensions among policy actors influence how policies are implemented. Lipsky contends that the gap between what policies set out to achieve and what happens in practice results because frontline providers exercise discretion in the way they implement, or ignore, policies, developing coping mechanisms to deal with difficult implementation environments. In this way, policy is effectively re-interpreted and such modifications may render the best intentioned policies ineffective [25]. Given the role of actors in policy implementation, we drew on “Street level bureaucracy” theory to guide our study [25]. This article explores the implementation of the VB policy in an Ecuadorian hospital to analyse the factors that affect effective implementation of intercultural health policies at the local level.

### Study setting

The hospital where the study took place provides emergency and short-stay outpatient and inpatient care in gynaecology and obstetrics, general medicine, paediatrics and general surgery. This hospital is located in a small town in the Ecuadorian Andes where 53% of the population are indigenous and 48% are mestizo [14]. A significant number of people, mostly indigenous people, do not have a formal employment but work in family business manufacturing and marketing traditional handcrafts [14].This has made this town relatively prosperous and it is argued that indigenous people have managed to lift themselves out of poverty without losing their cultural identity (Hurtado in [23]). This area has also been identified as the ‘intellectual cradle’ of the Ecuadorian indigenous movement which was one of the strongest in Latin America [23] and was critical in the emergence of the VB policy [27].

### Data collection

This research adopted a qualitative methodology and drew on the experiences and perspectives of actors involved in maternal health services in a local hospital in Ecuador. The principal investigator (AL) collected data from October 2009 to December 2010 (18 months after the VB was first implemented) through in-depth interviews. Interviews were conducted in Spanish by AL, a native speaker, and lasted 60 min on average.

Forty six respondents were selected for in-depth interviews using theoretical sampling and snowballing until saturation was reached and efforts were made to ensure the sample included respondents with different professional role, seniority, place of work, views on the VB policy, gender, and ethnic group. The sample included respondents working at the local hospital and surrounding primary health clinics as well as respondents working at the provincial and national level (Table 1). Respondents were interviewed in places where confidentiality could be maintained. Interviews were digitally recorded and transcribed. One respondent declined to be recorded and detailed notes were taken instead.

**Table 1:** Respondents interviewed by place of work and ethnic background

Respondent	Place of work			Ethnic background
	Local hospital	PHC clinics	Others	Indigenous/Mestizo
HCWs	13	6	0	0/19
Community leaders & key informants			7	6/1
Managers	4	4	4	3/9
TBAs	4			4/0
Policy-makers			4	1/3
Total	21	10	15	14/32

During fieldwork AL conducted participant observation too, which was carried out in the local hospital, a primary health clinic (PHC) clinic and an NGO clinic as well as during social events with community members and HWs. Data were also collected during a focus-group discussion with policy-makers, HWs, managers and indigenous community leaders that AL attended as an observer. In addition, detailed field notes were taken and relevant documents collected for analysis. Documents and field notes were used to inform interview guides, triangulate results and contextualise findings.

### Data analysis

AL coded and managed the data using Nvivo 8 and analysed it manually in Spanish. Quotations were translated by AL who is bilingual. Data analysis took place in two stages. During fieldwork we used elements of grounded theory; we took an iterative approach between data collection and analysis and we used inductive and deductive methods simultaneously [13, 16, 41]. Once data collection ended, we focused on analysing interview transcripts and field notes. We identified and described the key elements of respondents’ accounts. We compared and looked for relationships and associations between different themes to understand and interpret the VB policy implementation. We then carried out a comparative analysis of respondents’ accounts and looked for divergent cases to refine our analysis [16, 42]. Data derived from different methods of data collection were then triangulated as a way of clarifying conflicting information, providing a fuller picture of the research problem [41, 45] and enhancing reflexivity [45]. We also drew heavily on street-level bureaucracy theory to interpret the VB policy implementation. The data excerpts selected here are used to illustrate typical findings and/or eloquent explanations of the phenomena studied [16].

### Ethics approval and consent to participate

The main study received ethical approval from the ethics committee at the London School of Hygiene & Tropical Medicine and in Ecuador was approved by the ethics committee of the Universidad of Otavalo and other stakeholders, including the hospital directorate. Respondents were given information about the research and verbal or written consent was obtained prior to each interview. Interviews were digitally recorded when respondents consented; otherwise, detailed notes were taken.

## Results

A number of important issues emerged from the data regarding how the VB policy was negotiated and implemented in the hospital. First we describe the interpretations of the VB policy that different actors held. Next we explore the tensions between western and indigenous medical models of care, particularly the involvement of TBAs in hospitals. Finally, in the light of findings on interpretations and tensions we analyse the negotiations around policy implementation itself.

### Actors’ interpretations of the VB policy

For indigenous respondents, regardless of their professional role as HWs, managers, policy-makers, TBAs or community leaders, the VB policy was an expression of traditional medicine and as such a core element of their indigenous ethnic identity. Indigenous respondents stated that the VB policy implementation in the maternity department of a public hospital was an achievement of the indigenous movement because, after years of struggle, the State was starting to recognise traditional medicine as a valid medical system. The VB policy implementation was referred to by indigenous respondents as an opportunity to strengthen traditional medicine and indigenous identity as well as to advance indigenous right to health; ultimately, the VB policy meant a more equitable relationship between mestizo and indigenous culture.

For our community the vertical birth has been a thousand-year-old tradition but western medicine did not recognise it. They said that it damaged women’s health, that the vertical birth was very bad... Yet, we say that what [health professionals] do in hospitals is bad because they maltreat babies and women and they don’t provide adequate care (...). Now, the Ministry of Health has recognised an alternative medical system and for us it is very important because it shows that we are making advances in achieving our right to health. (Community leader, Indigenous).

Managers and policy-makers emphasised the current legal and policy framework that supported the VB policy and contrasted past and present health policy: Ecuador had gone through a *“dark neoliberal process”*, as one policy-maker put it, in which citizens’ rights were not guaranteed. The new Constitution passed in 2008 meant a break from the past as the State recognised its responsibility to fulfil citizens’ right to health and interculturallity as an overarching principle to inform health policy.

Whilst HWs also supported the VB policy goal, it brought into focus tensions between western and traditional medicine which led, initially, to considerable opposition. Most HWs were concerned about the clinical implications they attributed to the VB policy and the introduction of TBAs in the maternity department.

### Tensions between western and traditional medicine

#### Clinical complications attributed to the VB policy

Based on their initial experiences, HWs consistently reported that VB deliveries carried more risks than births in lithotomy position (with the patient lying on her back with her knees flexed and thighs apart). Several HWs argued that they never learned to conduct upright deliveries during their undergraduate training and that the doctor hired to train them up was incompetent. As these HWs explained, they observed how the trainer practised and they attributed the increase in obstetric complications to his poor aseptic technique. HWs thus felt unable to conduct VBs safely. HWs recalled how during the early implementation period there were various obstetric near-misses and neonatal deaths amongst women having a VB. Complications included post-partum haemorrhage resulting from an increase in vaginal tears and incomplete delivery of the placenta; an increase of dilatation and curettage (D&C) procedures; lack of asepsia, as HWs could not keep a sterile field because women constantly moved and contaminated the fields with excrements when they pushed, and the impossibility to perform episiotomies which they used to expedite or facilitate delivery and avoid vaginal tears, particularly in first-time mothers (primiparous). Interview and observation data suggest that these complications may have been caused by the way in which HWs managed upright deliveries. As some HWs pointed out:

At the beginning [of the VB implementation], perhaps because we were not very experienced, there was an increased incidence of vaginal tears amongst women who had a vertical birth, there were more D&C because there were retained placentas (Mestizo HW).

Furthermore, HWs stated that they lacked resources to deal with potential complications, chiefly uninterrupted access to theatre, and qualified staff to monitor labouring women. Therefore, HWs concluded that the VB was most suitable for low-risk women and women who had more than one child (multiparous women) and advised high-risk women (e.g. primiparous, slow labour) to have a lithotomy birth.

Apart from TBAs and managers, only two HWs spontaneously stated that upright birthing positions brought significant clinical benefits for the labouring woman. These respondents had experience with VBs during their early careers working in indigenous communities and were sensitive to the plight of indigenous women. One of them reported that she supported the VB policy precisely because of its clinical benefits.

How would we, as health professionals, be involved in something that we thought was bad for patients? But we thought it was good for [patients], we saw cases of breech presentation in which the woman would stand up and delivered easily. Then, how could I not support it? (Mestizo HW).

### Articulating traditional medicine and biomedicine

Of all the changes brought about by the VB policy, respondents’ accounts indicate that the most controversial one was the integration of TBAs on the labour ward which brought into focus tensions between traditional medicine and biomedicine.

TBAs and indigenous respondents (health managers and policy-makers) emphasised that TBAs’ role was to link the community and the hospital, advocate for women and protect them from HWs’ discrimination.

There is still discrimination in the [hospital], there are still HWs that don’t agree with TBAs and women who want a vertical birth. I recently had a problem with a nurse. The nurse was very rude to a patient; she was shouting at the patient: ‘that’s what [labour] is like, let me sleep!’. I stood next to the patient, I didn’t leave her side. [The patient] told me: ‘Thank God you are here!’ and I said: ‘We need to get through this; I’m not leaving your side’. I didn’t leave [the patient] until she delivered. I also told the nurse: ‘the patient is getting upset’ and the nurse answered: ‘I’m not a bad person, I’m just explaining to her how things are.’ (TBA).

Many HWs equated TBAs and traditional medicine with the VB and referred to them all as unscientific, potentially dangerous, obsolete and opposed to modern biomedicine.

We can’t say that TBAs are right because they practise according to their traditions, because we have science and science is based on evidence. (Mestizo HW).

HWs stated that since they were ultimately accountable for patients’ outcomes, it was their role to make clinical decision; TBAs should not conduct deliveries in hospital and should be supervised by them. Further, HWs believed that TBAs’ role should be limited to those aspects that they considered positive, such as linking the hospital and the community and supporting women in labour. A minority of HWs added that TBAs should not be in hospital at all and that TBAs were only appropriate in rural areas where there was no access to medical care. According to HWs, TBAs sometimes compromised patient care by contaminating sterile areas as TBAs were not familiar with aseptic techniques or gave women conflicting advice which could result in delaying emergency obstetric care and poor outcomes.

A HW recalled the case of a woman whose baby was breech and was advised by her doctor to have a caesarean section. The TBA however advised against it; she did a *‘manteo’* (tossing the patient in a blanket to turn the baby from breech into cephalic position) so the patient could have a vaginal delivery. The patient then left the hospital and returned after a few hours with the baby partially delivered; the baby’s head had got stuck in the birth canal and had died. Reflecting on this case, the HW stated that:

[Indigenous women] think that the best is to have a vaginal delivery because having a caesarean section implies that they can’t carry out their normal work... This is an understandable position but it becomes a barrier to accept a life-saving treatment. The TBA told this woman ‘I can do a manteo’... [the patient] came back five hours later which was lethal for the baby (Mestizo HW).

Some doctors and midwives also resented working alongside TBAs because they felt their skills, acquired through long years of study and work, were being levelled to those of unqualified and unskilled practitioners.

I respect TBAs but they don’t follow guidelines on asepsia. Then, why did we build a hospital? Why did we study eight, nine or even twelve years to become specialists? A hospital should be the cleanest place, particularly in theatre or the delivery room, everything is disinfected and then someone from the street, wearing street clothes comes and touches medical instruments. What are we talking about? So, it’s five-hundred years^1^ of medicine gone to waste; it’s useless! (Mestizo HW).

In spite of the criticisms that HWs levelled against TBAs, many HWs also identified several ways in which TBAs contributed to patient care. HWs, even those who did not fully support TBAs presence in the hospital, stated that they valued some TBAs skills such as giving massages, herbal remedies, the emotional support given to labouring women, their knowledge about homebirths and their ability to rotate foetuses in very specific cases. However, HWs said that TBAs’ most important contribution was linking the hospital and community and that the presence of TBAs increased indigenous women’s trust in the hospital.

I think TBAs play an important role, we could have even avoided the maternal death we had because [indigenous women] don’t come for fear of the hospital and doctors... but if [indigenous women] see a TBA they feel more confident. (Mestizo HW).

### Negotiating the VB policy implementation

At the time of data collection, the VB policy was reported by respondents to be an “established” and “successful” policy but our findings indicate that the initial stages of implementation were marked by intense conflict within the team. Managers, who supported the VB policy provided training and resources for clinical (e.g. upright delivery techniques) and non-clinical (e.g. interpersonal skills) components. They also capitalised on the support of political figures and institutions and made use of incentives (e.g. praising and extending contracts supporters of the VB policy) and sanctions (e.g. firing and side-lining detractors of the VB policy) to enforce implementation. Despite power discrepancies, HWs found creative strategies to resist or compromise the VB policy though managers ultimately succeeded in enforcing implementation as they were more powerful than HWs.

#### Capitalising on political and institutional support

Those respondents who backed the VB policy explained that the unwavering support given by political and MOH authorities at the national and local level was one of the main factors that enabled implementation. To respondents, this support was evidenced by the various visits that the Minister of Health herself and other senior MOH officials made to the hospital. The VB policy was firmly supported by the United Nations Population Fund (UNFPA), the local government and the indigenous community. The role of these actors in the VB policy is explored in detail elsewhere [27]. The hospital management team, led by an indigenous doctor as hospital director, was very committed to the VB policy. As implementation progressed, the VB policy gained supporters within the hospital. Elaborating on this a manager said:

When the opposition [to the VB policy] got too strong, it was made clear [to those who opposed the VB policy] that the hospital director was not alone; his decisions were supported by the [indigenous organization] and if [HWs] opposed the [VB policy], indigenous leaders would come to [the hospital] to demand explanations. There were even rumours that indigenous people would occupy the hospital (...). The provincial health director and the health sub-secretary at the national level gave us a lot of support (...). The national health sub-secretary told [the management team]: ‘if anything happens, if [HWs] don’t comply, you call me directly’. (Indigenous Manager).

#### Influencing perceptions on the VB policy: use (and misuse) of data

According to interview data both parties sought to influence people’s perceptions of the VB policy by challenging its safety and uptake amongst patients and in this way gain further support or opposition to it.

HWs’ initial experiences conducting VBs had raised serious concerns about its safety and they were keen on collecting data to assess VBs health outcomes. However, HWs selectively sampled medical histories of VB patients, particularly those cared for by the (unpopular) trainer, looking for evidence of complications such as vaginal tears and incomplete delivery of the placenta. As a respondent pointed out, the objective was to discredit the trainer and therefore, the VB policy.

VB supporters manipulated quantitative data too: a HW who was involved in various obstetric emergencies of women having VBs expressed her disbelief when managers produced a report in which no obstetric complications from VB policy were reported. Managers argued that a random sample of three cases had been selected but the number of cases (*n* = 50) was so small that the entire sample could have been taken according to this HW.

[The report results] were not true, there were lots of complications. Why don’t [managers] tell the truth? (...) [Managers] tried to make it look as if the VB was wonderful but it wasn’t true, at the beginning it was very difficult. (Mestizo HW).

The uptake of VBs amongst patients was another issue raised by supporters and detractors to shape perceptions of the VB policy. For example, supporters portrayed the VB policy as a success because they claimed it had resulted in a 9% increase in access to hospital maternity services, mostly indigenous women from rural areas. However, hospital data were not routinely disaggregated by ethnic origin or place of residence. Furthermore, hospital data show that the number of hospital deliveries followed an upward trend since at least 2004 and that the increase in use of hospital services was mirrored by other specialities where intercultural health policies had not been introduced.

To affect VBs uptake, interview data show that both parties sought to allocate staff who agreed with their position to the labour ward. In this way, managers hired, extended the contracts of HWs willing to support the VBs policy implementation or allocated them to the labour ward and sanctioned those HWs who opposed it. For example, various respondents brought up the case of a HW who reportedly lost her job because she had refused to perform a VB and was rude to a patient. Another case cited by respondents was that of a doctor whose contract was reportedly terminated after he presented a study showing a higher incidence of complications amongst women having a VB and recommended the discontinuation of the VB policy. Whether or not these were the actual reasons why managers fired these HWs, HWs reported increasing their compliance with the VB policy as they feared losing their jobs. In turn, interview data show that HWs tried to reduce the uptake of VBs amongst patients by refusing to conduct VBs altogether, reallocating team members who supported the VB policy to outpatients, misreporting VBs as lithotomy births and not helping illiterate TBAs to document their input in patient care.

#### Changing HWs’ attitudes and behaviours towards indigenous patient

Research findings suggest that the implementation of the VB policy contributed to shaping HWs’ attitudes and behaviours towards indigenous women in a number of ways. According to respondents, in the past discrimination against indigenous women in the health service was commonplace and evidenced by long waiting times and HWs’ rudeness. This resulted in inequitable access to services and health outcomes for indigenous women.

[Health] professionals were quite rude to [indigenous] patients; for instances, once they told an [indigenous] woman: ‘You must have a tubal ligation!’ in such a way that [health professionals] were imposing it (…) using inappropriate and unprofessional words. [Indigenous] patients felt coerced, they complained about maltreatment and didn’t want to go to the hospital. (Mestizo HW).

However, respondents noted that the VB policy had contributed to shaping HWs attitudes towards indigenous women.

They used to say that before [the VB policy] there was maltreatment, discrimination because doctors insulted patients, they would tell [indigenous patients] off... but we are young doctors and we don’t agree with [maltreating patients]. We try to be the kindest and most understanding with the patient. (Mestizo HW).

As part of the VB policy, the new management team actively sought to increase HWs’ accountability and monitor their behaviour by supporting TBAs when they reported HWs rudeness towards women and making use of patients’ feedback. Managers operated an *“open doors”* policy to encourage patients’ feedback. HWs and managers noticed a surge in indigenous patients’ complaints (usually about long waiting times and HWs rudeness) as they felt *“empowered”* when an indigenous doctor became hospital director*.*

[Indigenous] patients had trust in us; they could come to the director’s office (...) and tell us about the problems in the hospital such as that [HWs] didn’t want to see them and that they had to wait very long. (Indigenous Manager).

Several HWs received verbal and written memos asking them to clarify an incident. Often managers were satisfied with HWs’ explanations but applied sanctions if they felt HWs were at fault. To avoid sanctions, several HWs reported improving their behaviour towards indigenous women.

Managers and policy-makers agreed that staff attitudes had improved substantially but several respondents warned that historical trends were slow to reverse. In the words of a respondent:

The main challenge is still how [HWs] treat indigenous users (...). Ecuador is a country with a long history of colonialism, discrimination and exclusion of indigenous peoples. This is clearly demonstrated by the way State services such as health and education are delivered. Indigenous peoples were considered invisible and therefore government employees never thought to guarantee their rights. You can easily make cultural adaptations in hospitals but if HW attitudes don’t change then you have the same discrimination in [hospitals] with vertical birth rooms. (Mestizo Policy-maker).

## Discussion

Lipsky argues that the job of street-level bureaucrats (SLB) is highly scripted to achieve policy objectives and that despite having a strong sense as service workers, SLB, often cannot perform to the highest standard for each individual case due to lack of resources. In trying to manage their difficult jobs, SLB exercise discretion and develop coping mechanisms that narrow the gap between their ideals and reality [25]. A number of studies have used Lipsky’s theory to explore HWs response to policies in the public health sector in low and middle income countries [39, 48] and our study adds to this growing body of literature.

Our study revealed that HWs developed coping mechanisms not in response to lack of resources but in response to a clash of values. Our results show that while HWs supported the values enshrined by the VB policy (i.e. promoting ethnic equity) they also believed that VBs led to obstetric complications, which run counter to professional values of non-maleficence and acting on the patient’s best interest.

The introduction of TBAs in the maternity service was arguably the most contentious aspect of the VB policy. HWs resented working alongside TBAs as they felt that their skills were being levelled against those of unqualified indigenous women, and that TBAs represented and promoted a medical system perceived as *“unscientific”* and *“backwards”.* HWs dealt with this tension by redefining the TBA’s role. They did not allow TBAs to deliver babies in hospital. Instead, HWs allocated TBAs jobs they considered limited the risks TBAs posed to patients (e.g. cleaning), and jobs that maximized benefits for the patients or did not interfere with their own jobs (e.g. acting as doulas, translating and linking the community and the hospital).

Another coping mechanism used by HWs to deal with the VB policy was to define the eligibility for VBs. HWs allowed multiparous low-risk women to have VBs but actively discouraged primiparous women from having one because HWs considered them at higher risk of developing complications and needing an episiotomy, which could not be performed in upright positions. HWs also advised women to have a lithotomy birth when labour was considered high-risk or not progressing adequately (e.g. slow head descent).

We found that HWs’ perceptions and discretionary responses to the VB policy restricted women’s choice for VBs and limited TBAs’ role in hospital. At the same time, these strategies allowed HWs to restore some degree of control over their jobs and cope with the pressure they were under to implement the VB policy. HWs were thus de facto policy-makers, as Lipsky’s theory suggests, but they operated within the constraints imposed by managers, confirming findings by other researchers [1, 25]. Managers appeared to accept HWs’ reinterpretation of the VB policy as a compromise to enable implementation to go ahead, even if the policy was modified as a result. That is, policy implementation resulted from an ongoing *negotiation* between HWs and managers.

Another way in which HWs in our study narrow the gap between their ideals and the reality is by providing their best care for a subset of the population and neglecting others they considered less worthy. In establishing who is worthy or unworthy of their best care, HWs draw on social prejudices and stereotypes, and in doing so reflect the value that the State and society place on different people. Historically, indigenous people across the world have been largely marginalized and disadvantaged by State services [4, 7, 31, 40]. Indigenous women in Ecuador for example have reported degrading treatment in health services and receiving substandard care (e.g. being shouted at, being left alone in active labour). This has kept indigenous women from accessing health services which has negative implications for maternal and neonatal health outcomes [26]. Entrenched ethnic discrimination in health services is difficult to overcome and many intercultural health policies have failed because they have neglected this critical aspect [31]. In this sense, the greatest contribution of the VB policy was to tackle head-on HWs’ discriminatory attitudes towards indigenous women. We identified the following enabling factors:

First, managers set HWs clear expectations of behaviour towards indigenous women. Managers monitored closely HWs’ behaviour through patient’s feedback. Patients’ complaints were mainly motivated by long waiting times and HWs degrading treatment which were seen by indigenous people as evidence of ethnic discrimination in the health service. Managers responded to complaints on a case-by-case basis, demanding explanations from HWs and applying sanctions as they saw fit. Managers monitored HWs’ behaviour through TBAs too. TBAs, as insiders, knew the hospital’s inner workings and, although significantly less powerful than HWs, they developed subtle strategies to get better care for their patients without fuelling an outright conflict. TBAs would, for instance, remind HWs of the patients’ needs and would physically stand next to their patients. HWs believed that TBAs had the managers’ support and would mould their behaviour to avoid sanctions.

Second, managers used incentives and sanctions to facilitate implementation. For example, managers praised HWs who displayed positive attitudes towards indigenous women and extended their contracts. Managers also sanctioned HWs whose behaviour fell below expected standards. For instances, the reported dismissal of a HW who refused to conduct VBs increased compliance with the VB policy as HWs feared losing their jobs. Managers also drew on the continuous support of national and local policy-makers, and the indigenous community to make it clear that implementation was not a matter of choice but mandatory and that dissent with the policy would be sanctioned.

Through these interventions of managers, HWs had to reflect on how their attitudes influenced indigenous women’s access to health services and outcomes. In doing so, HWs were able to align their professional and personal values with those of the policy and to recognize their critical role in guaranteeing indigenous women’s right to health. The importance of reflective practice has also been noted by other researchers [29].

Our results demonstrate that actors’ professional and personal values influenced how they interpreted, promoted or resisted and eventually delivered the VB policy. In this way, the study confirms an extension of Lipsky’s theory identified by Aniteye and Mayhew [1] who found that providers values, not only resource constraints as proposed by Lipsky, play a critical role in shaping actors’ responses to policies. Our findings also confirm the adjustments Lipsky himself made to his framework, that the management level also plays a critical role in frontline interpretation of policy [12, 25].

Our findings have a number of practice implications. Despite HWs reservations, the evidence shows that some interventions promoted by TBAs are supported by scientific evidence. For example, mobilising during the first stage of labour (dilatation) reduces its length [24]. Likewise, upright positions during the second stage (pushing) are associated with a shorter duration, less assisted deliveries, less episiotomies, less severe pain and fewer abnormal foetal heart rate patters but an increase in second degree tears and estimated blood loss [17]. Intake of light food during labour has not been found to increase the risk of vomiting or to influence obstetric and neonatal outcomes [37]. The use of routine episiotomy, as performed by HWs in Otavalo, is also associated with more severe perineal trauma and more healing complications. Most importantly, performing routine episiotomies do not prevent those complications they intend to avoid (i.e. severe perineal trauma, painful sexual intercourse, or urinary incontinence) [6]. Finally, continuous support for women in labour (e.g by a relative, a HW or a TBA) has meaningful clinical benefits for mothers and babies such as more normal delivers, less assisted births and caesarean sections, less use of analgesia, shorter labours and less need for neonatal resuscitation [18]. Current national maternal clinical guidelines [35] are consistent with international studies though this evidence was ignored by HWs. Given HWs palpable interest in practising “modern” medicine, portraying the VB as a policy that promotes evidence-based interventions (as well as ethnic health equity) could be used as a lever to facilitate implementation. This would require training HWs on research methodology and its application to clinical practice. It is also plausible that since the goal of the VB policy was difficult to criticise for it ultimately promoted ethnic equity, some HWs may have referred to the lack of scientific basis of the VB policy as an acceptable and non-sanctionable rationale to express their dissent with the policy.

While integrating TBAs in the health service was very contentious, as it has been also noted by other researchers in Ecuador [11], our findings demonstrate that TBAs can have a positive effect in addressing discrimination in the health service and supporting particular evidence-based interventions in maternal care [18, 24, 35]. Furthermore, international studies have found that integrating TBAs in the health service improves the use of skilled birth attendant, perinatal mortality and possibly maternal mortality [2, 5, 20, 50]. Nonetheless, TBAs can also jeopardise women’s health and this needs to be addressed; our study found that in some cases TBAs’ advice resulted in delaying life-saving interventions. In this context TBAs have an important role to play to improve maternal and neonatal health but it should not involve making clinical decisions. Instead, their role in hospitals should be to promote the evidence-base interventions aforementioned (e.g. supporting labouring women) and work in partnership with HWs. Providing a space where HWs and TBAs can communicate respectfully and having clearly defined agreed roles could ease the tensions that may arise when two different medical systems are articulated and support policy implementation.

## Conclusions

Our study has shown that intercultural health policies such as the VB policy have the potential to facilitate indigenous women’s access to maternity services and improve maternal and neonatal health outcomes. These findings are particularly important given the current emphasis many Latin American countries place on promoting intercultural health policies.

Based on our findings we conclude that promoting indigenous women’s access to health services should involve more than token cultural adaptations to maternity services. Addressing discriminatory attitudes towards indigenous women should be at the core of any intercultural health policy; managers are key players in facilitating these changes.

Finally, on a theoretical level, and consistent with research elsewhere, this study shows that Lipsky’s street-level bureaucracy theory is useful to analyse policy implementation in low and middle income countries. This study confirms an extension of Lipsky’s theory whereby providers’ values, not only resource constraints, shape actors’ responses to policies and that *negotiation* between HWs and managers enables policy implementation.

## Abbreviations

D&C: Dilatation and curettage
HWs: Health workers
MOH: Ministry of health
NGO: Non-govermental organization
PHC: Primary health care
SLB: Street-level bureaucrats
TBAs: Traditional birth attendants
UNFPA: United Nations population fund
VB: Vertical birth

## Footnotes

1 Referring to 500 years since the Spanish colonisation of South America.
